# Clinical Significance of Pleural Effusion in *Mycoplasma pneumoniae* Pneumonia in Children

**DOI:** 10.3390/pathogens10091075

**Published:** 2021-08-25

**Authors:** Seo-Hee Kim, Eun Lee, Eun-Song Song, Yun-Young Lee

**Affiliations:** 1 Department of Pediatrics, Chonnam National University Hospital, Gwangju 61469, Korea; shkim2968@gmail.com (S.-H.K.); essong@jnu.ac.kr (E.-S.S.); 2 Department of Pediatrics, Chonnam National University Medical School, Gwangju 61469, Korea; 3 Department of Radiology, Chonnam National University Hospital, Gwangju 61469, Korea; yunyoung0219@gmail.com

**Keywords:** children, *Mycoplasma pneumoniae*, pleural effusion, pneumonia

## Abstract

The clinical significance of pleural effusion in *Mycoplasma pneumoniae* (MP) pneumonia in children has not yet been elucidated. Herein, we investigated the clinical implications of pleural effusion in children with MP pneumonia. Overall, 150 children with MP pneumonia transferred to a tertiary hospital were enrolled in this study. Information on their clinical, laboratory, and radiological features was retrospectively obtained from medical chart reviews. In total, 24 (16.0%) children had pleural effusion at the time of admission. The duration of fever and length of hospitalization were significantly longer in the pleural effusion group than in the non-pleural effusion group. A significantly higher proportion of individuals in the pleural effusion group had a poor response to stepwise treatment for MP pneumonia. The mean C-reactive protein, lactate dehydrogenase, and aspartate aminotransferase levels were significantly higher in the pleural effusion group than in the non-pleural effusion group at admission. The prevalence of severe pneumonia, defined on the basis of the extent of pneumonic lesions on chest radiography, was higher in the pleural effusion group than in the non-pleural effusion group. However, there was no significant intergroup difference in the proportion of macrolide-resistant MP cases or respiratory viral coinfections. The presence of pleural effusion in children with MP pneumonia indicated a more severe clinical course and poor treatment response. The results of the present study would help in the creation of a therapeutic plan and prediction of the clinical course of MP pneumonia in children.

## 1. Introduction

*Mycoplasma pneumoniae* (MP) is one of the most common causes of community-acquired pneumonia in children [1]. MP infections present as cyclic epidemics every 3–7 years, depending on the geographic location [2]. MP infection is considered a benign or self-limiting disease; however, the prevalence of refractory MP pneumonia, characterized by persistent fever and/or disease progression despite appropriate treatment with antibiotics and immunomodulators, has been increasing [3,4,5]. The prevalence of macrolide-resistant MP (MRMP) pneumonia has also been increasing [6]. Owing to the absence of a cell wall in MP, antibiotics effective against MP infections are limited. The combination of these factors makes it challenging to treat MP pneumonia and reduce its complications.

To improve the clinical outcomes of MP pneumonia, previous studies aimed to determine the predictive factors for clinical course, treatment response, and prognosis [7]. In addition, radiological findings combined with clinical characteristics and laboratory findings can be useful in predicting the clinical course and treatment response in children with MP pneumonia. However, studies on the clinical implications of chest radiographic findings are limited [8]. Pleural effusion, one of the complications of community-acquired pneumonia, can occur during the clinical course of MP pneumonia [9,10,11,12,13]. A previous study reported that MP was the causative pathogen in 19% of the cases of complicated parapneumonic effusion and empyema [12]. Another study reported that MP pneumonia requiring intensive care unit admission in children was associated with a higher prevalence of pleural effusion than that not requiring intensive care unit admission (65% vs. 10%, *p* < 0.001) [10]. However, studies comparing the clinical features and radiological and laboratory findings according to the pleural effusion status in MP pneumonia are lacking; the availability of such information would be helpful in identifying the clinical significance of pleural effusion in children with MP pneumonia. Therefore, the present study investigated the clinical implications of pleural effusion in children with MP pneumonia by comparing the clinical, laboratory, and radiological features of MP pneumonia in relation to the pleural effusion status.

## 2. Materials and Methods

### 2.1. Study Population

This study enrolled 150 children with MP pneumonia who were admitted to Chonnam National University Hospital between May 2019 and March 2020. All the children were previously healthy, without any chronic respiratory diseases, except eight children with physician-diagnosed asthma. Among the total population diagnosed with MP pneumonia, 16.0% (*n* = 24) had pleural effusion on chest radiographs at the time of admission; the patients with pleural effusion were referred from other hospitals. The clinical manifestations and the laboratory and radiological findings were retrospectively obtained through medical chart reviews.

MP pneumonia was defined using the following three criteria: (1) acute respiratory symptoms, including fever, cough, and/or sputum; (2) proven MP infection based on the results of MP-specific IgM using blood samples and polymerase chain reaction (PCR) analysis of the sputum samples; and (3) abnormal chest radiograph and/or abnormal findings on physical examination, including abnormal breathing sounds. The presence of pleural effusion was diagnosed based on chest radiography findings [14,15]. The local Institutional Review Board (IRB) approved this study and waived the need for informed consent because of the retrospective study design (IRB No. CNUH-2019-261).

### 2.2. Definition

Severity of pneumonia was defined based on the extent of pneumonic lesions on chest radiography as follows: mild, pneumonic lesions in less than one-fourth of the total lung field; moderate, pneumonic lesions in more than one-fourth but less than one-third of the total lung field; and severe, pneumonic lesions in more than one-third of the total lung field. All the patients were treated using stepwise treatment for MP pneumonia as follows: (1) initial treatment with macrolides, preferably azithromycin (10 mg/kg/day, once orally for 3 days), regardless of the macrolide resistance of MP, and 1–2 mg/kg/day (maximum 30 mg/dose) intravenous methylprednisolone with consideration of the treatment provided by the previous clinic; (2) when fever was persistent or the clinical signs and symptoms showed deterioration despite treatment with macrolides and low-dose systemic corticosteroids, ciprofloxacin or tetracyclines were introduced in cases of MRMP pneumonia; and (3) if patients responded poorly to the second-line treatment for MRMP cases or first-line treatment for macrolide-sensitive MP pneumonia cases, 10–15 mg/kg/day methylprednisolone pulse therapy was administered for three consecutive days with close observation for adverse effects associated with high-dose steroid therapy [16,17]. Treatment response was determined as “good” when the patients showed improvement on physical examination or chest radiography within 2–3 days after admission to our hospital. A “slow response” was defined as a slight improvement of respiratory symptoms and/or chest radiography findings within 1 week but not within 2–3 days of stepwise treatment of MP pneumonia. “No response or progression” was defined as no improvement or progression of respiratory symptoms and/or chest radiography findings even after 1 week of stepwise treatment for MP pneumonia [15]. Patients in the good and slow response groups were classified as the “response group” and those with no response to treatment or progression of MP pneumonia were classified as the “poor response group”. 

### 2.3. Microbiological Studies

All the patients had a confirmed MP infection based on the results obtained with a *Mycoplasma pneumoniae* real-time PCR kit (Biocore, Seoul, South Korea), which analyzed the enrolled patients’ sputum samples [18], and of the serologic tests for MP-specific IgM using the LIAISON MP IgM kit (DiaSorin, Dublin, Ireland) [15]. Macrolide resistance was evaluated by the identification of point mutations at sites 2063 or 2064 in domain V of 23S rRNA using the *Mycoplasma pneumoniae* real-time PCR kit (Biocore, Seoul, South Korea).

The sputum samples were analyzed for *Streptococcus pneumoniae*, *Haemophilus influenzae*, MP, *Chlamydia pneumoniae*, *Bordetella pertussis*, and *Legionella pneumophila* using the Seeplex PneumoBacter detection kit (Seegene Inc., Seoul, South Korea) to access the presence of respiratory bacterial coinfections [19].

### 2.4. Statistical Analysis

Binary logistic regression analysis was performed to identify the factors associated with the development of pleural effusion in MP pneumonia, selected based on the significant difference and/or factors considered clinically important. Adjustments were made for age and sex. All the statistical analyses were performed using SPSS version 25.0 for Windows (SPSS, Inc., Chicago, IL, USA). *p*-values < 0.05 were considered significant.

## 3. Results

### 3.1. Demographic Characteristics of the Study Population

No significant differences were observed in the demographic characteristics between those with and those without pleural effusion except age at the time of diagnosis of MP pneumonia (Table 1). The mean age of the patients without pleural effusion (mean, 5.7 years; standard deviation (SD), 3.8 years) was lower than that of those with pleural effusion (mean, 7.3 years; SD, 3.4 years). Age can affect the manifestations of MP pneumonia in children. Therefore, we summarized the baseline characteristics of the study population after classifying the study population with regard to the age of 6. 

### 3.2. Comparison of Clinical Manifestations According to the Pleural Effusion Status

The duration between the onset of MP pneumonia symptoms and admission as well as the total fever duration were significantly longer in the children with pleural effusion than in those without pleural effusion (Table 2). The need for supplemental oxygen was more common in the pleural effusion group (*n* = 4/24, 16.7%) than in the non-pleural effusion group (*n* = 5/126, 4.0%) (*p* = 0.016). The proportion of the poor response group was significantly higher in the pleural effusion group than in the non-pleural effusion group (*p* = 0.005). There was no significant intergroup difference in the duration between symptom onset and initiation of antibiotic therapy.

The treatment with antibiotics or a systemic corticosteroid was started earlier in the MP pneumonia with pleural effusion group than in the MP pneumonia without pleural effusion group, though the difference was not statistically significant. Defervescence occurred earlier in the MP pneumonia with pleural effusion group after a systemic corticosteroid therapy (*p* = 0.028). Defervescence also occurred earlier in the MP pneumonia with pleural effusion group after antibiotic therapy was administered, though the difference was not statistically significant. No cases complicated by pleural effusion required additional procedures for its removal.

### 3.3. Comparison of Laboratory Findings at the Time of Admission by the Pleural Effusion Status

The levels of C-reactive protein (CRP), lactate dehydrogenase (LDH), and aspartate aminotransferase (AST) at the time of admission were significantly higher and the level of lymphocytes (%) was significantly lower in the pleural effusion group than in the non-pleural effusion group (Table 3).

### 3.4. Comparison of the Microbiological Characteristics by the Pleural Effusion Status

There was no significant difference in the macrolide sensitivity of MP by the pleural effusion status (Table 4). *Haemophilus influenzae* and *Streptococcus pneumoniae* were two of the most commonly coinfected respiratory bacteria in the children with MP pneumonia. However, *Chlamydia pneumoniae* was not identified in any cases of MP pneumonia in this study. The prevalence of respiratory viral coinfection was not associated with the presence of pleural effusion in children with MP pneumonia.

### 3.5. Comparison of Radiological Findings by the Pleural Effusion Status

The proportion of severe pneumonia cases, defined by the extent of pneumonic lesions on the chest radiograph, was higher in the pleural effusion group than in the non-pleural effusion group (Table 5).

### 3.6. Factors Associated with Pleural Effusion in Children with MP Pneumonia

Older age, longer duration of fever during the illness, higher CRP and LDH levels, and lower lymphocyte (%) levels were associated with the development of pleural effusion in MP pneumonia (Table 6). In addition, oxygen supplementation, severity of pneumonia, and a lack of response to stepwise treatment for MP pneumonia were significantly associated with pleural effusion in MP pneumonia.

## 4. Discussion

In this study, we found that the presence of pleural effusion in children with MP pneumonia suggests a more severe clinical course of MP pneumonia, reflected by a higher need for oxygen support, prolonged hospitalization and fever, poor response to stepwise treatment, increased extent of pneumonic infiltration, and abnormal blood test results, including those for LDH and CRP. However, macrolide resistance and respiratory viral coinfection were not associated with pleural effusion in children with MP pneumonia. Based on the results of the present study, prediction of the clinical course of MP pneumonia according to the presence of pleural effusion would be helpful in therapeutic planning for better outcomes in children with MP pneumonia.

Pleural effusion is one of the most common causes of transfer to tertiary hospitals in patients with MP pneumonia. The prevalence of pleural effusion in MP pneumonia can differ according to the characteristics of the study population. Previous studies have reported that the prevalence of pleural effusion in MP pneumonia is 20.3–20.7% [20,21], although these studies included small sample sizes. In this study, pleural effusion was observed in 16% of the pediatric MP pneumonia cases referred to our tertiary hospital. Pleural effusion in all the patients investigated in this study resolved following stepwise treatment of MP pneumonia without additional procedures regardless of the extent of pleural effusion. Considering the results of this study, pleural effusion alone might not be troublesome in the clinical course of MP pneumonia. However, a case of MP pneumonia complicated by pleural effusion might suggest a severe clinical course with poor response to MP pneumonia treatment.

Although the exact pathophysiology underlying pleural effusion in MP pneumonia has not yet been well-identified, direct invasion, continuum of the MP infection, or exaggerated immune responses might be associated with its development in patients with MP pneumonia [9]. A previous study suggested that pleural effusion caused by MP infection can be classified into two patterns: one characterized by no detection of the MP genome and lower levels of cytokines, including interleukin 18 and interleukin 8, and the other, characterized by persistent chest disease with detection of the MP genome and higher levels of interleukin 18 and interleukin 8 [22]. In this study, we could not classify the characteristics of pleural effusion because the procedures to identify the characteristics of or drain the pleural effusion were not performed. Pleural effusion that complicated the MP pneumonia cases in this study resolved gradually with the early application of immunomodulatory drugs, such as systemic corticosteroids, which were administered as part of the stepwise treatment for MP pneumonia. The earlier defervescence observed in the MP pneumonia with pleural effusion group than in the no pleural effusion group after the administration of a systemic corticosteroid suggests that exaggerated immune responses might be associated with the development of pleural effusion in MP pneumonia. Further studies are needed to elucidate the pathophysiology of pleural effusion in MP pneumonia.

Although some studies reported predictive factors for a poor response to treatment in cases of MP pneumonia such as LDH, interleukin 18, and ferritin levels [15,23,24], to date, no study has focused on the clinical significance of pleural effusion in MP pneumonia. A previous study investigated the characteristics of pleural effusion in MP pneumonia in children and proved the presence of MP in the pleural effusion using PCR [9]. In that study, the detection of MP in the pleural effusion was associated with delayed resolution of abnormal chest radiographic findings in children with MP pneumonia [9]. In our study, the presence of pleural effusion in MP pneumonia was associated with the involvement of more higher extent of pneumonic lesions on chest radiographs at the time of admission. Based on the previous and present studies, the presence of pleural effusion in MP pneumonia might suggest the severe extent and prolonged lesions on chest radiographs.

Respiratory viral coinfection could be one of the causes for the development of pleural effusion in MP pneumonia cases; however, this study found no significant difference in the proportion of patients with respiratory viral coinfection in the groups with and without pleural effusion. Moreover, there was no significant difference in respiratory bacterial coinfection between the MP pneumonia with pleural effusion group and the MP pneumonia without pleural effusion group, although PneumoBacter PCR for the identification of respiratory bacterial coinfection was performed in only 48.7% (*n* = 73/150) of the cases. Furthermore, we identified that the macrolide resistance status of MP did not affect the occurrence of pleural effusion. This study identified the microbiological characteristics of sputum samples in children with MP pneumonia complicated by pleural effusion. Since there is some debate on the effects of respiratory viral and/or bacterial coinfection on the clinical course of MP pneumonia in children [25,26], further large sample-sized studies are needed to investigate the effects of respiratory viral and/or bacterial coinfection in MP pneumonia with pleural effusion.

This study had some limitations. First, it included only patients with MP pneumonia treated at one tertiary hospital, most of whom had been transferred from primary or secondary medical centers due to complications of MP pneumonia. This suggests the selection bias in the enrolled population because more severe cases were enrolled in this study. However, pleural effusion is common in more severe cases of MP pneumonia; therefore, the results of this study are applicable to real clinical situations. More multicenter studies are necessary to confirm the results of this study, which could help improve the prognosis of MP pneumonia in children in the era of increasing macrolide resistance of MP and refractory MP pneumonia.

## 5. Conclusions

This study identified that pleural effusion in MP pneumonia is associated with a more severe clinical course, poorer treatment response, and severe pneumonia based on the extent of pulmonary lesions, although pleural effusion in all the assessed cases resolved without the need for additional procedures. However, macrolide resistance and respiratory viral coinfection did not affect the occurrence of pleural effusion in the children hospitalized with MP pneumonia. The results of this study could help clinicians predict the clinical course and treatment responses of MP pneumonia in children.

## Figures and Tables

**Table 1:** Baseline characteristics of the study population.

	Variables, *n* (%)	Pleural Effusion (−)	Pleural Effusion (+)	*p*-Value
children <6 years of age	N	71 (91.0)	7 (9.0)	NA
	Age at diagnosis of MP pneumonia, mean ± SD, years	3.1 ± 1.4	3.7 ± 1.1	0.259
	Male, *n* (%)	35/71 (49.3)	3/7 (42.9)	0.529
	Referred cases, *n* (%)	66/71 (93.0)	7/7 (100.0)	0.617
	Presence of allergic diseases, *n* (%)	33/71 (46.5)	3/7 (42.9)	0.587
	Atopic dermatitis, *n* (%)	1/71 (1.4)	0/7 (0.0)	0.910
	Allergic rhinitis, *n* (%)	32/71 (45.1)	3/7 (42.9)	0.615
	Asthma, *n* (%)	6/71 (8.5)	0/7 (0.0)	0.558
children ≥6 years of age	N	55 (76.4)	17 (23.6)	NA
	Age at diagnosis of MP pneumonia, mean ± SD, years	9.2 ± 3.1	8.7 ± 3.0	0.502
	Male, *n* (%)	25/55 (45.5)	9/17 (52.9)	0.396
	Referred cases, *n* (%)	55 (100.0)	17 (100.0)	NA
	Presence of allergic diseases, *n* (%)	36/55 (65.5)	13/17 (76.5)	0.295
	Atopic dermatitis, *n* (%)	2/55 (3.6)	0/17 (0.0)	0.581
	Allergic rhinitis, *n* (%)	34/55 (61.8)	12/17 (70.6)	0.361
	Asthma, *n* (%)	10/55 (18.2)	2/17 (11.8)	0.420

MP, *Mycoplasma pneumoniae*; N, number; NA, not applicable; SD, standard deviation.

**Table 2:** Comparison of clinical characteristics according to the presence or absence of pleural effusion in children with MP pneumonia.

Variables, *n* (%) or Mean ± SD	Pleural Effusion (−)	Pleural Effusion (+)	*p*-Value
Duration between the symptom onset and admission, days	6.1 ± 4.0	10.4 ± 4.8	0.026
Duration of fever during the illness, days	6.4 ± 3.7	8.6 ± 4.0	<0.001
Hemoptysis	3/126 (2.4)	0/24 (0.0)	0.445
Fever	125/126 (99.2)	24/24 (100.0)	0.661
Oxygen supplementation	5/126 (4.0)	4/24 (16.7)	0.016
ICU admission	0/126 (0.0)	0/24 (0.0)	NA
Need for mechanical ventilation	0/126 (0.0)	0/24 (0.0)	NA
Response to the treatment			0.005
Response	114/125 (91.2)	17/24 (70.8)	
Poor response	11/125 (8.8)	7/24 (29.2)	
Total duration of hospitalization, days	8.2 ± 4.0	15.4 ± 7.1	<0.001
Duration between the symptom onset and start of antibiotic therapy, days	3.6 ± 3.4	3.4 ± 3.5	0.816
Duration between the symptom onset and start of systemic corticosteroid therapy, days	7.4 ± 5.3	6.5 ± 4.1	0.377
Duration between the start of antibiotic therapy and defervescence, days	4.0 ± 4.0	2.7 ± 3.2	0.152
Duration between start of systemic corticosteroid therapy and defervescence, days	1.2 ± 2.7	0.4 ± 0.7	0.028
Duration between the symptom onset and admission to our hospital, days	6.5 ± 3.8	7.6 ± 3.6	0.123

ICU, intensive care unit; NA, not applicable; SD, standard deviation.

**Table 3:** Comparison of laboratory findings according to the presence or absence of pleural effusion in the children with MP pneumonia.

Variables, Mean ± SD (Range)	Pleural Effusion (–) (*n* = 126)	Pleural Effusion (+) (*n* = 24)	*p*-Value
WBC, ×10^3^/μL	9300 ± 4700	9300 ± 4400	0.934
Neutrophils (%)	62.2 ± 14.0	67.2 ± 18.2	0.130
Lymphocytes (%)	26.7 ± 11.9	19.5 ± 10.7	0.007
Eosinophils (%)	1.8 ±2.6	2.1 ± 2.0	0.536
Monocytes (%)	8.6 ± 3.8	7.7 ± 3.3	0.282
CRP, mg/dL	2.3 ± 3.2	8.2 ± 7.8	0.002
ESR, mm/h	35.4 ± 19.5	40.6 ± 21.0	0.271
Procalcitonin, ng/dL	0.2 ± 0.4	0.4 ± 0.5	0.230
LDH, IU/L	751.1 ± 280.2	1107.8 ± 507.2	0.003
AST, IU/L	40.0 ± 27.7	74.3 ± 64.5	0.017
ALT, IU/L	29.9 ± 35.7	54.5 ±57.4	0.053
Albumin, g/dL	5.3 ± 1.5	5.2 ± 1.7	0.866
MP IgM at admission, index	4.3 ± 3.3	5.3 ± 3.3	0.194

AST, aspartate aminotransferase; ALT, alanine aminotransferase; CRP, C-reactive protein; ESR, erythrocyte sedimentation rate; LDH, lactate dehydrogenase; MP, *Mycoplasma pneumoniae*; SD, standard deviation; WBC, white blood cells.

**Table 4:** Comparison of microbiologic characteristics according to the presence or absence of pleural effusion in children with MP pneumonia.

Variables, *n* (%) or Mean ± SD (Range)	Pleural Effusion (–)	Pleural Effusion (+)	*p*-Value
Macrolide sensitivity			0.173
MSMP	9/124 (7.3)	0/24 (0.0)	
MRMP	115/124 (92.7)	24/24 (100.0)	
Coinfection with respiratory viruses	51/122 (41.8)	12/23 (52.2)	0.357
Number of coinfected respiratory viruses	0.5 ± 0.7	0.7 ± 0.9	0.413
Rhinovirus coinfection	28/126 (22.2)	8/24 (33.3)	0.243
Adenovirus coinfection	18/126 (14.3)	1/24 (4.2)	0.172
Bacterial coinfection identified using PneumoBacter PCR	23/59 (39.0)	5/14 (35.7)	0.264
*Haemophilus influenzae*	8/59 (13.6)	4/14 (28.6)	
*Streptococcus pneumoniae*	7/59 (11.9)	0/14 (0.0)	
Both *Haemophilus influenzae* and *Streptococcus pneumoniae*	8/59 (13.6)	1/14 (7.1)	

MP, *Mycoplasma pneumoniae*; MRMP, macrolide-resistant *Mycoplasma pneumoniae*; MSMP, macrolide-sensitive *Mycoplasma pneumoniae*; PCR, polymerase chain reaction; SD, standard deviation.

**Table 5:** Comparison of the radiological findings according to the presence or absence of pleural effusion in children with MP pneumonia.

Variables, *n* (%)	Pleural Effusion (−)	Pleural Effusion (+)	*p*-Value
Severity of pneumonia at admission based on the chest radiograph			<0.001
Mild	16/126 (12.7)	0/24 (0.0)	
Moderate	90/126 (71.4)	8/24 (33.3)	
Severe	20/126 (15.9)	16/24 (66.7)	
Trend (*p*)	<0.001		
Characteristics on the chest radiograph at the time of admission			<0.001
Lobar consolidation	22/126 (17.5)	16/24 (66.7)	
Patchy consolidation	59/126 (46.8)	7/24 (29.2)	
Peribronchial infiltration	34/126 (27.0)	1/24 (4.2)	
Diffuse nodular opacity	3/126 (2.4)	0/24 (0.0)	
Diffuse infiltration	8/126 (6.3)	0/24 (0.0)	
Development of PTE	2/126 (1.6)	2/24 (8.3)	0.060
Development of PIBO	15/126 (11.9)	3/24 (12.5)	0.934

PTE, pulmonary thromboembolism; PIBO, postinfectious bronchiolitis obliterans.

**Table 6:** Factors associated with the development of pleural effusion in children with MP pneumonia.

Variables	aOR *	*p*-Value
Age, years	1.131 (1.007–1.271)	0.038
Duration of fever during the illness, days	1.196 (1.080–1.323)	0.001
Oxygen supplementation at the time of admission	6.023 (1.426–25.439)	0.015
Severity of pneumonia based on the chest radiograph at admission		
Mild and moderate	Ref.	
Severe	10.465 (3.899–28.090)	<0.001
Lymphocytes (%)	0.945 (0.900–0.991)	0.021
CRP, mg/dL	1.221 (1.106–1.348)	<0.001
LDH, IU/L	1.003 (1.001–1.004)	<0.001
Mycoplasma IgM at the time of admission, index	1.127 (0.981–1.296)	0.091
Response to MP pneumonia treatment		
Response	Ref.	
Poor response	4.789 (1.577–14.549)	0.006
Total duration of hospitalization, days	1.133 (1.041–1.232)	0.004

* Adjusted by age and sex; aOR, adjusted odds ratio; CRP, C-reactive protein; LDH, lactate dehydrogenase; NA, not applicable; Ref., reference.

## Data Availability

Please refer to the suggested Data Availability Statements in section “MDPI Research Data Policies” at https://www.mdpi.com/ethics (accessed on 1 August 2021).
